# Candida lusitaniae Peritonitis Associated With Nonsurgical Pneumoperitoneum: A Case Report Exploring a Causal Relationship or Coincidence

**DOI:** 10.7759/cureus.112257

**Published:** 2026-07-08

**Authors:** Mia M Shokry, Luke Ciulla, Christian Przeslawski, Lianne Caceres, Wiley Fan, Michael Rebock

**Affiliations:** 1 Medicine, Michigan State University College of Osteopathic Medicine, East Lansing, USA; 2 General Surgery, Corewell Health Farmington Hills Hospital, Farmington Hills, USA; 3 Infectious Disease, Corewell Health Farmington Hills Hospital, Farmington Hills, USA

**Keywords:** acute abdomen, candida lusitaniae, exploratory laparotomy, fungal peritonitis, iatrogenic pneumoperitoneum, idiopathic spontaneous pneumoperitoneum, micafungin, nonperforative pneumoperitoneum, nonsurgical pneumoperitoneum, suprapubic catheter placement

## Abstract

Pneumoperitoneum is classically associated with perforated abdominal viscus and typically prompts emergent surgical evaluation and intervention. However, a small subset of patients present with nonsurgical pneumoperitoneum in the absence of gastrointestinal perforation, creating a significant diagnostic and management challenge for clinicians. Distinguishing surgical from nonsurgical causes is particularly difficult when patients demonstrate signs of peritonitis, as the presence of abdominal tenderness, guarding, or systemic inflammatory findings may strongly suggest bowel perforation despite negative imaging or operative findings. In rare cases, infectious etiologies may further complicate the clinical picture. *Candida lusitaniae* peritonitis is exceptionally uncommon outside the setting of peritoneal dialysis or significant immunocompromise and has rarely been described in association with nonsurgical pneumoperitoneum. We report an unusual case of pneumoperitoneum without gastrointestinal perforation complicated by *C. lusitaniae* peritonitis in an immunocompetent patient following recent suprapubic catheter placement. Fungal peritonitis due to *C. lusitaniae *is exceptionally uncommon, especially in patients without a history of peritoneal dialysis, abdominal surgery, or significant immunocompromise. Recognition of these atypical presentations is important to avoid diagnostic delays and to guide appropriate medical and surgical management strategies.

## Introduction

Pneumoperitoneum, characterized by the presence of free intraperitoneal gas, is a clinical finding that classically signals a perforated abdominal viscus in an estimated 90% of cases [1]. This typically necessitates urgent surgical intervention. A rare but clinically relevant subset of patients can be classified as having “spontaneous” or “nonsurgical” pneumoperitoneum. This poses a diagnostic challenge, with free gas present without evidence of visceral perforation. This phenomenon has a diverse range of etiologies, including intrathoracic air dissection, in which air is commonly spread to the peritoneum intravascularly; gynecological entry via the fallopian tubes; or idiopathic causes, in which the gas is absorbed without sequelae. It is important that this subset of patients be recognized to avoid unnecessary invasive laparotomy procedures in patients who may be otherwise successfully managed conservatively. We present a case of pneumoperitoneum in the absence of perforation complicated by the presence of *Candida lusitaniae* peritonitis.

## Case presentation

**Figure 1:**
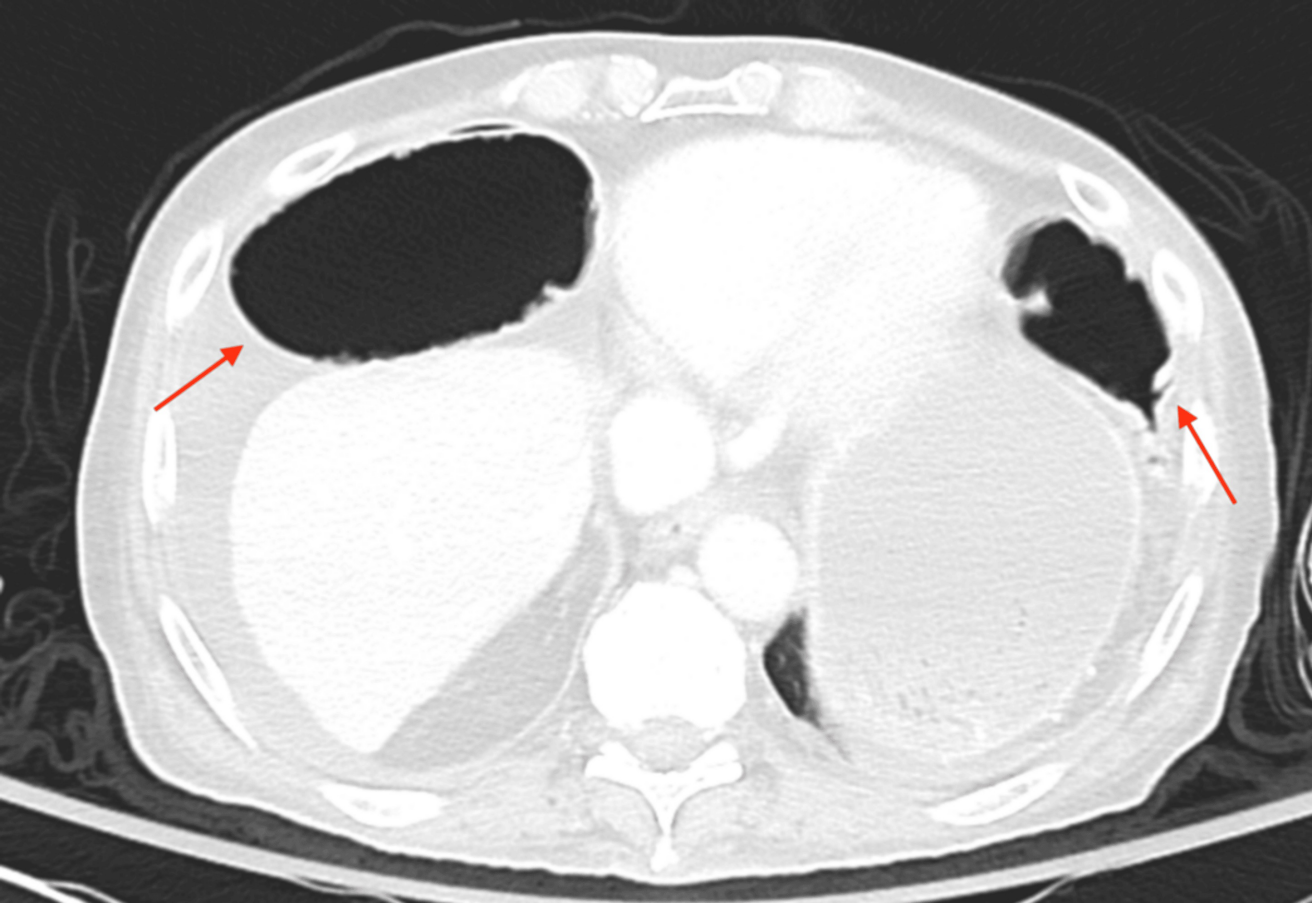
Axial upper cut of CT abdomen and pelvis with IV contrast showing intraperitoneal air on the left and right of heart consistent with perforated viscus (red arrows). Trace ascites are noted CT: computed tomography

An 80-year-old man with a medical history significant for essential hypertension, chronic kidney disease stage 3a (estimated glomerular filtration rate 45-49 mL/minute), heart failure with reduced ejection fraction (stage D), paroxysmal atrial fibrillation, pulmonary hypertension, hyperlipidemia, and chronic urinary retention presented four days after cystoscopy with right retrograde pyelogram, bilateral ureteral stent exchange, and suprapubic catheter placement. He presented with diminished urine output, abdominal pain, and altered mental status. He had no history of immunocompromise, malignancy, chronic immunosuppressive therapy, or peritoneal dialysis. Computed tomography (CT) of the abdomen and pelvis demonstrated free intraperitoneal air concerning for perforated viscus, although no definite source of perforation was identified radiographically (Figure 1).

On arrival at the emergency department, he was hypotensive with a blood pressure of 99/53, with vital signs otherwise normal. Physical examination revealed a distended, firm abdomen with diffuse tenderness, guarding, rigidity, and positive rebound tenderness. Initial laboratory findings on admission are presented in Table 1. Notably, the white blood cell count was within normal limits at 6.6 x 10^9^/L, with inflammatory markers showing elevated lactic acid at 3.8 mmol/L and ferritin at 2,198 ng/mL. Urinalysis showed the presence of yeast.

**Table 1:** Initial laboratory results Elevated lactic acid and ferritin levels were noted, along with mild anemia and thrombocytopenia. Reference ranges are those provided by the reporting laboratory

Laboratory	Result	Reference range
Lactic acid	3.8 mmol/L	0.0-2.0 mmol/L
White blood cell count	6.6 × 10^9^/L	3.5-10.1 × 10^9^/L
Red blood cell count	3.6 × 10^12^ /L	4.31-5.48 × 10^12^/L
Hemoglobin	11.5 g/dL	13.5-17.0 g/dL
Mean corpuscular volume	99.2 fL	79.5-100.4 fL
Platelet count	130 × 10^9^/L	150-400 × 10^9^/L
C-reactive protein	1.9 mg/L	<8.0 mg/L
Ferritin	2,198 ng/mL	14-338 ng/mL

Following CT results and the patient’s clinical exam, he was taken for an emergent exploratory laparotomy, which demonstrated no evidence of a perforated viscus. The patient was found to have purulent peritonitis and underwent intraoperative abdominal washout. Fungal cultures obtained from intraoperative peritoneal fluid grew *C. lusitaniae*. Antifungal susceptibility testing was not performed. The patient completed a two-week course of intravenous micafungin with clinical improvement and was discharged 15 days following admission. Micafungin was selected because echinocandins are recommended as first-line therapy for invasive candidiasis and provide reliable activity against *C. lusitaniae*, a species recognized for its ability to develop resistance to amphotericin B. Antifungal susceptibility testing was not performed in this case. At outpatient follow-up with General Surgery eight days following discharge, he reported complete resolution of abdominal pain and was recovering without evidence of recurrent infection. A summary of the patient's clinical course is provided in Table 2.

**Table 2:** Timeline of the patient's clinical course from initial hospitalization through outpatient follow-up CT: computed tomography

Date	Clinical course
19 Days prior to admission	Admitted for acute cystitis with hematuria and urinary retention
4 Days prior to admission	Underwent cystoscopy with right retrograde pyelogram, bilateral ureteral stent exchange, and suprapubic catheter placement. Discharged later that day
Day 1	Presented to the emergency department with diminished urine output, abdominal pain, and altered mental status. Physical examination demonstrated diffuse abdominal tenderness with guarding, rigidity, and rebound tenderness. CT abdomen/pelvis demonstrated free intraperitoneal air concerning for perforated viscus
Day 1	Emergent exploratory laparotomy performed. No gastrointestinal perforation was identified. Purulent peritonitis was encountered, and intraoperative abdominal washout was performed. Empiric intravenous vancomycin and meropenem were initiated postoperatively
Day 3	Intravenous micafungin was initiated for suspected fungal peritonitis. (Verify date)
Day 4	Intraoperative peritoneal fluid fungal cultures grew Candida lusitaniae. Infectious Diseases consultation recommended continuation of micafungin and discontinuation of vancomycin. Meropenem was discontinued following culture results. Antifungal susceptibility testing was not performed
Day 15	Completed a two-week course of intravenous micafungin and was discharged home in stable condition
8 Days after discharge	Outpatient follow-up with General Surgery demonstrated complete resolution of abdominal pain without evidence of recurrent infection

## Discussion

This article describes the presentation of pneumoperitoneum without perforation, complicated by *C. lusitaniae* peritonitis. Whether *C. lusitaniae* peritonitis contributed to the development of pneumoperitoneum remains uncertain. The association observed in this case should be considered hypothesis-generating rather than evidence of causation. An alternative and perhaps more plausible explanation is recent suprapubic catheter placement, which may have introduced air into the peritoneal cavity during instrumentation. The fungal peritonitis may therefore represent either an independent process or a secondary finding identified during operative exploration.

Prior documented cases of *C. lusitaniae* peritonitis are almost exclusively associated with peritoneal dialysis, with the incidence of this pathogen being a rare cause of peritonitis overall. In this case, *C. lusitaniae* presents in the presence of a suprapubic catheter in a patient with no history of peritoneal dialysis. Although *C. lusitaniae* peritonitis has rarely been reported outside the setting of peritoneal dialysis, published cases have generally occurred in patients with severe intra-abdominal pathology or significant immunocompromise. Reports involving immunocompetent patients without these traditional risk factors remain exceptionally uncommon, highlighting the unique presentation described in this report.

Pneumoperitoneum may often present alongside peritonitis, typically attributed to Gram-negative enteric flora. However, management may be complicated by the isolation of rare pathogens, such as *C. lusitaniae*. This pathogen is most commonly associated with fungemia in 80% of reported cases, with peritonitis and other intra-abdominal infections being much less frequently described [2]. *C. lusitaniae* peritonitis is most commonly associated with peritoneal dialysis [3]. Additional key risk factors for invasive *C. lusitaniae* infection include immunocompromised states (particularly hematologic malignancies with neutropenia), total parenteral nutrition, prior abdominal surgery, central venous catheters, and prior broad-spectrum antibiotic exposure [2,4,5]. Although our patient had several chronic medical conditions, he had no evidence of immunocompromise or prior peritoneal dialysis, making this presentation particularly unusual. Recent urologic instrumentation and suprapubic catheter placement may have predisposed the patient to both intra-abdominal contamination and nonsurgical pneumoperitoneum. Among Candida peritonitis cases, only one of 109 was *C. lusitaniae*, compared with *Candida* albicans (58%), *Candida* glabrata (20%), and *Candida* krusei (8%) [6]. It presents a therapeutic challenge relative to other species as it often presents with rapidly acquired resistance to amphotericin B [4,7]. Intraperitoneal candidiasis carries a high mortality risk with rates up to 40%, particularly when presented in the setting of peritonitis [8,9].

With fungal peritonitis already representing a minority of cases, the detection of *C. lusitaniae* in an immunocompetent patient not undergoing peritoneal dialysis demonstrates a unique case in the setting of pneumoperitoneum. Despite the detection of *C. lusitaniae* on culture, the underlying etiology of pneumoperitoneum in this case remains unclear. The presence of a suprapubic catheter may represent a source of potential iatrogenic-induced introduction of air into the peritoneum.

Identifying potential sources of pneumoperitoneum outside the setting of perforation may help determine which patients can be managed conservatively. While nonsurgical pneumoperitoneum is typically defined as the absence of intra-abdominal perforation, laparoscopy may still be indicated in the presence of peritonitis, as suggested by elevated white cell count and inflammatory markers. In patients with pneumoperitoneum complicated by peritonitis, operative management has been associated with improved outcomes in appropriately selected patients [10]. In this case, diffuse abdominal tenderness with guarding, rigidity, rebound tenderness, and an elevated serum lactate raised strong concern for significant intra-abdominal pathology despite the absence of leukocytosis or elevated C-reactive protein. Given these findings, operative exploration remained appropriate despite the eventual absence of gastrointestinal perforation. Intraoperative washout and peritoneal fluid cultures subsequently identified the underlying fungal infection and guided targeted antifungal therapy.

## Conclusions

This case highlights a rare presentation of nonsurgical pneumoperitoneum complicated by Candida lusitaniae peritonitis in an immunocompetent patient without a history of peritoneal dialysis. Although the temporal association between fungal peritonitis and pneumoperitoneum is notable, this report does not establish a causal relationship between the two entities. Recent suprapubic catheter placement represents a plausible alternative explanation for the pneumoperitoneum observed in this patient. Nevertheless, this case emphasizes that patients with pneumoperitoneum and clinical signs of peritonitis continue to warrant prompt operative evaluation even when gastrointestinal perforation is ultimately absent. Intraoperative cultures may identify uncommon pathogens that direct appropriate antimicrobial therapy, and clinicians should maintain a broad differential diagnosis when evaluating atypical presentations of pneumoperitoneum.
